# Metagenomic analysis provides bases on individualized shift of colon microbiome affected by delaying colostrum feeding in neonatal calves

**DOI:** 10.3389/fmicb.2022.1035331

**Published:** 2022-11-01

**Authors:** Yang Song, Shubo Wen, Fuyong Li, Amanda Fischer-Tlustos, Zhixiong He, Le Luo Guan, Michael Steele

**Affiliations:** ^1^Animal Nutrition and Feed Science, College of Animal Science and Technology, Inner Mongolia Minzu University, Tongliao, China; ^2^Department of Agriculture, Food and Nutritional Sciences, University of Alberta, Edmonton, AB, Canada; ^3^Key Laboratory of Zoonose Prevention and Control at Universities of Inner Mongolia Autonomous Region, Tongliao, China; ^4^Department of Infectious Diseases and Public Health, Jockey Club College of Veterinary Medicine and Life Sciences, City University of Hong Kong, Kowloon, Hong Kong SAR, China; ^5^Department of Animal Biosciences, University of Guelph, Guelph, ON, Canada; ^6^Key Laboratory of Agro-ecological Processes in Subtropical Region, Institute of Subtropical Agriculture, Chinese Academy of Sciences, Changsha, China

**Keywords:** delayed colostrum, neonatal calf, colon microbiome, metagenome, individual variance

## Abstract

This study investigated the effect of colostrum feeding time on the colon digesta microbiome of 2-day-old dairy calves using whole-genome-based metagenome sequencing, aiming to understand the dynamic changes of the colon microbiome when the colostrum feeding is delayed. In total, 24 male Holstein calves were grouped to different pasteurized colostrum feeding time treatments randomly: TRT0h (45 min after birth, *n* = 7); TRT6h (6 h after birth, *n* = 8); and TRT12h (12 h after birth, *n* = 9). Bacteria, archaea, eukaryotes, and viruses were identified in the colon microbiome, with bacteria (99.20%) being the most predominant domain. *Streptococcus*, *Clostridium*, *Lactobacillus*, *Ruminococcus*, and *Enterococcus* were the top five abundant bacteria genera. For colon microbiome functions, 114 Kyoto Encyclopedia of Genes and Genomes (KEGG) pathways were identified, with nutrients metabolism-related functions “carbohydrate metabolism,” “amino acid metabolism,” “metabolism of cofactors and vitamins,” “metabolism of terpenoids and polyketides,” and “metabolism of other amino acids” being the top five secondary level of KEGG hierarchy functions. When colon microbiomes were compared, they were not affected by delaying first colostrum feeding at both taxonomic and functional levels. However, distinct clusters of colon microbiome profiles were shown based on PERMANOVA analysis despite of different colostrum feeding treatment, suggesting the individualized responses. Moreover, the relative abundance of microbial taxa, microbial functions, and differentially expressed genes was compared between the two distinct clusters, and different relationships were observed among host differentially expressed genes, differential levels of microbial taxa, and microbial functions between the two clusters. Our results suggest that the host may play an important role in shaping the colon microbiome of neonatal dairy calves in response to the early life feeding management. Whether the observed colon microbiome shifts affect gut health and function in the long term requires further research.

## Introduction

Calves are born without passive immunity because the maternal placenta separates the blood supply between the dam and fetus during pregnancy (Godden, 2008). Therefore, feeding immunoglobulin-rich colostrum, which is the first milking after calving, is of great importance to neonatal calves. Colostrum contains numerous nutrients, bioactive factors, and immunoglobulins, which play a fundamental role in ensuring the survival and passive immunity of newborn calves (Lopez and Heinrichs, 2022). The apparent efficiency of absorption (AEA) of immunoglobulins decreased from 30.5% to 15.8% when calves were fed colostrum within 1 h compared to those fed 12 to 18 h after birth (Osaka et al., 2014). More recently, Fischer et al. (2018) reported that delaying colostrum feeding from 1 to 12 h results in a decrease in AEA from 51.8% to 35.1%, further corroborating that calves should be fed colostrum immediately after birth.

Newborn calves have an immature GIT at birth, which is quickly colonized by maternal and environmental microbiota in the intestinal tract (DiGiulio, 2012; Aagaard et al., 2014; Weese and Jelinski, 2017; O'hara et al., 2020). Colostrum also contains vast variety of microbe that could affect early intestinal microbial colonization of neonatal calves (Yeoman et al., 2018). However, the pasteurization process is commonly used which could eliminate most of the bacteria in the colostrum. It has been suggested that delaying pasteurized colostrum feeding also influences plasma parameters (Hammon et al., 2000), ileum and colon mucosa-associated bacterial groups (Fischer et al., 2018; Ma et al., 2019), as well as microbial profiles and functions in the ileal digesta of neonatal calves (Song et al., 2021). To date, previous studies have mainly focused on the effect of colostrum feeding time on the transfer of passive immunity, as well as its impact on small intestinal microbial and host functional development of neonatal calves. Few attempts have been made to understand how colostrum feeding time influences the large intestinal digesta-associated microbial colonization and function.

For neonatal calves, colon is the major site of microbial colonization before ruminal development (Song et al., 2018), additionally, colon is related to host energy metabolism, health, immune system modulation, and physiological development (He et al., 2018; Song et al., 2018; Ma et al., 2019; Guo et al., 2021). Recent studies have determined the dynamic changes of the colon bacterial community of Holstein and crossbred calves in early life. Dias et al. (2018) found the relative abundance of *Oscillospira* and *Ruminococcus* genera in the colon remained high and unchanged during the preweaning period of crossbred calves. While Song et al. (2018) reported that digested-associated *Lactobacillus* and *Bacteroides* were the most predominant bacteria in the colon of Holstein dairy claves. Additionally, the effects of pre-weaning feeding management on programming colon microbial colonization have also been reported. It was suggested that the supplementation of low concentrations of antibiotics (Yousif et al., 2018), butyrate-fortified milk replacer (O'hara et al., 2018), as well as direct-fed microbials in the milk replacer, influence the colonization of digesta-associated microbiota in the colon (Fomenky et al., 2018; Samarasinghe et al., 2021). However, these studies only focused on bacterial compositional changes, and the functional shift of the colon microbiome in response to colostrum feeding management in neonatal calves has not been studied. Therefore, the objectives of this study were to characterize colon microbial composition and functional changes under different colostrum feeding time using metagenomic analysis, and to evaluate whether the colonic microbiome shift could be affected by individual differences of neonatal calves.

## Materials and methods

### Calves and colostrum feeding

The animal trail was conducted at the research farm (Dairy Research and Technology Centre) of the University of Alberta (Edmonton, Canada), following the procedures provided by The Livestock Care Committee of the University of Alberta (AUP00001595). In total, 24 Holstein bull calves were selected for the study, with birth body weights (BW) ranging from 35 to 50 kg. All the calves were removed from the dam immediately after birth to avoid contamination. Calves were then dried, ear tagged, treated with navel disinfectant, and housed in individual hutches with straw bedding.

All calves were randomly grouped into one of three treatments: colostrum feeding at 45 min (TRT0h, *n* = 7), 6 h (TRT6h, *n* = 8), or 12 h (TRT12h, *n* = 9) after birth. The pasteurized colostrum (62 g of IgG per L; Saskatoon Colostrum Company Ltd.; Saskatoon, SK, Canada) was fed to the calves at a rate of 7.5% BW at their respective time point. Then, all the calves received milk replacer meals (protein: 26%, fat: 18%; Excel Pro-Gro Calf Milk Replacer, Grober Nutrition, Cambridge, ON, Canada) at 2.5% BW every 6 h of life starting at 12 h after colostrum feeding.

### Colon digesta collection

The sample collection process was the same as described by Fischer et al. (2018). Briefly, all calves were euthanized at 51 h of age followed by colon digesta sample collection. The colon sample segment was defined as 30 cm distal to the ileocecal junction (Malmuthuge et al., 2019). The digesta contents were squeezed out into a 50-ml Falcon tube, and immediately frozen in liquid nitrogen and then stored at −80°C.

### DNA isolation

Genomic DNA was extracted from colon digesta according to the modified DNA extraction method (Yu and Morrison, 2004). Briefly, the frozen falcon tubes with colon digesta (~0.5 g) were thawed at 4°C the day before DNA extraction process. The digesta were resuspended in 1 ml of cell lysis buffer and were subjected to the BioSpec Mini-BeadBeater 8 (BioSpec, Bartlesville, OK), then cells were mechanically disrupted at 4,800 rpm for 3 min. After bead beating, lysed cells were incubated (70°C, 15 min) and the supernatant was collected for Genomic DNA precipitation. After precipitation, the DNA was purified using QIAamp Fast DNA Stool Mini Kit (QIAGEN Inc. CA, United States). DNA quantity and quality were measured using a NanoDrop 1,000 spectrophotometer (NanoDrop Technologies, Wilmington, DE, United States).

### Metagenomic sequencing and analysis of colon microbiome

After DNA extraction, the libraries of each sample for shot-gun whole-genome-based metagenome sequencing were constructed using the TruSeq DNA PCR-free library preparation kit (Illumina, CA, United States) according to the manufacturer’s instructions. Then, the quantity of each library was assessed using a Qubit 2.0 Fluorometer (Thermo Fisher Scientific, MA, United States), and sequenced at McGill University and Génome Québec Innovation Centre (Montreal, QC, Canada) using Illumina HiSeq 4000 (2 × 100 bp paired-end reads).

The detailed procedures of colon metagenome data analysis were described by Song et al. (2021). Quality control was performed to remove low-quality bases (quality scores < 30) and short reads (<75 bp) using fastq-mcf (Aronesty, 2013). Then, the eligible reads were mapped to the bovine genome (UMD 3.1) using TopHat 2 (threads:6, version 2.0.9; Kim et al., 2013) to remove host DNA contamination. To evaluate colon microbiome functional profiles, the filtered DNA sequences from each sample was assembled to contigs using MetaVelvet (Namiki et al., 2012) with a kmer size of 51. The identical contigs were binned from the pooled contigs of all the samples, and unique contigs were constructed. Then, Prodigal program was applied for gene prediction on the purpose of identifying protein-coding sequences (Hyatt et al., 2010). Unique predicted genes (≥100 bp) were preserved for the following analysis. The contigs were annotated against the KEGG database (Kanehisa et al., 2012) with UBLAST program (using the following parameters: E-value ≤ 1e − 5, bit score ≥ 60, sequence identity ≥ 30%), and then the qualified reads of each sample were aligned to annotated contigs for functional annotations. The relative abundance of the KEGG pathways of each colon sample was evaluated through the HUMAnN2 program (Abubucker et al., 2012). Meanwhile, the CPM of each pathway was calculated with the following formula: CPM = (number of reads mapped to a gene) ÷ (total number of reads mapped to all annotated genes) × 10^6^. The taxonomic analysis of colon digesta microbiome was performed using Metagenomic Rapid Annotations using Subsystems Technology (MG-RAST) version 3.3.9. The microbial taxa were assigned to phylum, family, and genus levels referencing to Refseq database, following e-value ≤ 1e − 5, identity ≥ 60%, and alignment ≥ 50 bp (Meyer et al., 2008).

### Short chain fatty acids measurement in the colon of newborn calves

Detailed procedures were the same as described previously (Guan et al., 2008). In this study, colon digesta samples (~ 0.1 g) were dissolved in 25% phosphoric acid solutions in a 5 ml tube, at a ratio of 4:1 according to volume. Acetate, propionate, butyrate, isobutyrate, isovalerate, and valerate concentrations were measured using gas chromatography (GC) with Short chain fatty acids (SCFA) concentrations presented as μmol/g fresh weight of digesta sample.

### Colonic transcriptome profiles analysis between calves classified into different groups based on metagenomes

Colon tissue transcriptome profiles were retrieved from our previous publication (He et al., 2018) and the profiles were compared between calves that were grouped based on their metagenomes (Group A and Group B). The levels of expression of genes between groups A and B in the colon tissue were compared. Log2 fold change of each gene was calculated using the equation: log2 fold change = log2(average cpm of Group A/average cpm of Group B). The threshold for differential expressed genes (DE genes) was defined using the following cut-off, with *p* < 0.05, log2 fold change ≤ −1 and ≥1, with positive values indicating upregulated in group A, and negative values indicating downregulated genes in group A. In addition, the unique genes and commonly expressed genes in groups A and B were plotted with a Venn Diagram. The DE genes and unique genes in Groups A and B were subjected to Annotation, Visualization, and integrated Discovery (DAVID) for up_keywords, gene ontology (Go) terms annotation and Kyoto Encyclopedia of Genes and Genomes (KEGG) pathways analysis, with enrichment score > 1, and *p*-value < 0.05 defined as significant.

### Statistical analyses

Microbial taxa, SCFAs concentration and molar proportion, the phenotypic parameters retrieved from our previous studies, including the concentration of plasma glucagon-like peptide (GLP-1), glucagon-like peptide (GLP-2), insulin (Inabu et al., 2018), and serum IgG (Fischer et al., 2018), the copy number of total bacteria, the abundance of *Clostridium* cluster XIVa, *Faecalibacterium prausnitzii*, and *E. coli*, as well as the transcriptomic profiles of colon tissues (He et al., 2018) were analyzed using R version 4.1.3 and SPSS 26.0 packages (IBM Corp., Armonk, NY). Microbial taxa with the relative abundance > 0.05% and present in more than half of the total animals within each treatment (TRT0h, TRT6h, or TRT12h) were considered as detected microbial taxa. Additionally, the metabolic pathways with CPM > 5 in at least 50% of the animals in each treatment were defined as detected microbial functions. The detected microbial taxa and functions were further analyzed in the downstream analysis. To identify the differences of bacterial and archaeal composition, as well as SCFAs concentration and molar proportion among three treatments, the non-parametric Kruskal–Wallis test was applied, and Dunn’s test was used to test the difference between any two treatment groups. The *p*-value was adjusted with Benjamini–Hochberg method for false discovery rate (FDR; Yoav and Yosef, 1995), with *p* < 0.05, and 0.05 ≤ *p* < 0.10 declared as statistical significance and tendency, respectively. Additionally, the abundance of colon microbiome functions was compared using linear discriminant analysis (LDA) effect size (LEfse; Segata et al., 2011), and features with LDA score > 2 and *p* < 0.05 were considered to be significantly different (Mottawea et al., 2016). Bacterial and archaeal general communities among different treatments were analyzed using Bray–Curtis dissimilarity matrices-based principal coordinate analysis (PCoA). Microbial metabolic functions were analyzed with principal component analysis (PCA), and PERMANOVA analysis was conducted to test the statistical difference among treatments. The original phenotypic data from the same animal trial in previous publications were obtained for deep comparison between calves with two type of colon metagenomes (groups A and B) using Welch’s *T*-test. Spearman’s rank correlation was performed between the varied microbial taxa, functions, and phenotypic parameters (butyrate concentration and DE genes) of groups A and B, with significance declared at |ρ| > 0.8 and *p*-value < 0.01.

## Results

### Colon digesta metagenomes of neonatal calves

In total, 744,939,525 reads were generated, and 378,452,001 reads were retained after quality control, with 15,768,833 ± 1,976,165 reads (mean ± SEM) per calf for colon microbiome. After subjecting the predicted genes to the KEGG database for functional annotation, 692,370 genes were successfully annotated. Then, the metagenome of each calf was mapped to the reference comprised of the above-annotated genes to define colon microbiome function. In total, 19.18% ± 5.64% (mean ± SEM) metagenomic sequences of each calf were mapped (Supplementary Dataset 1).

### Microbial functions in the colon of neonatal calves

**Figure 1:**
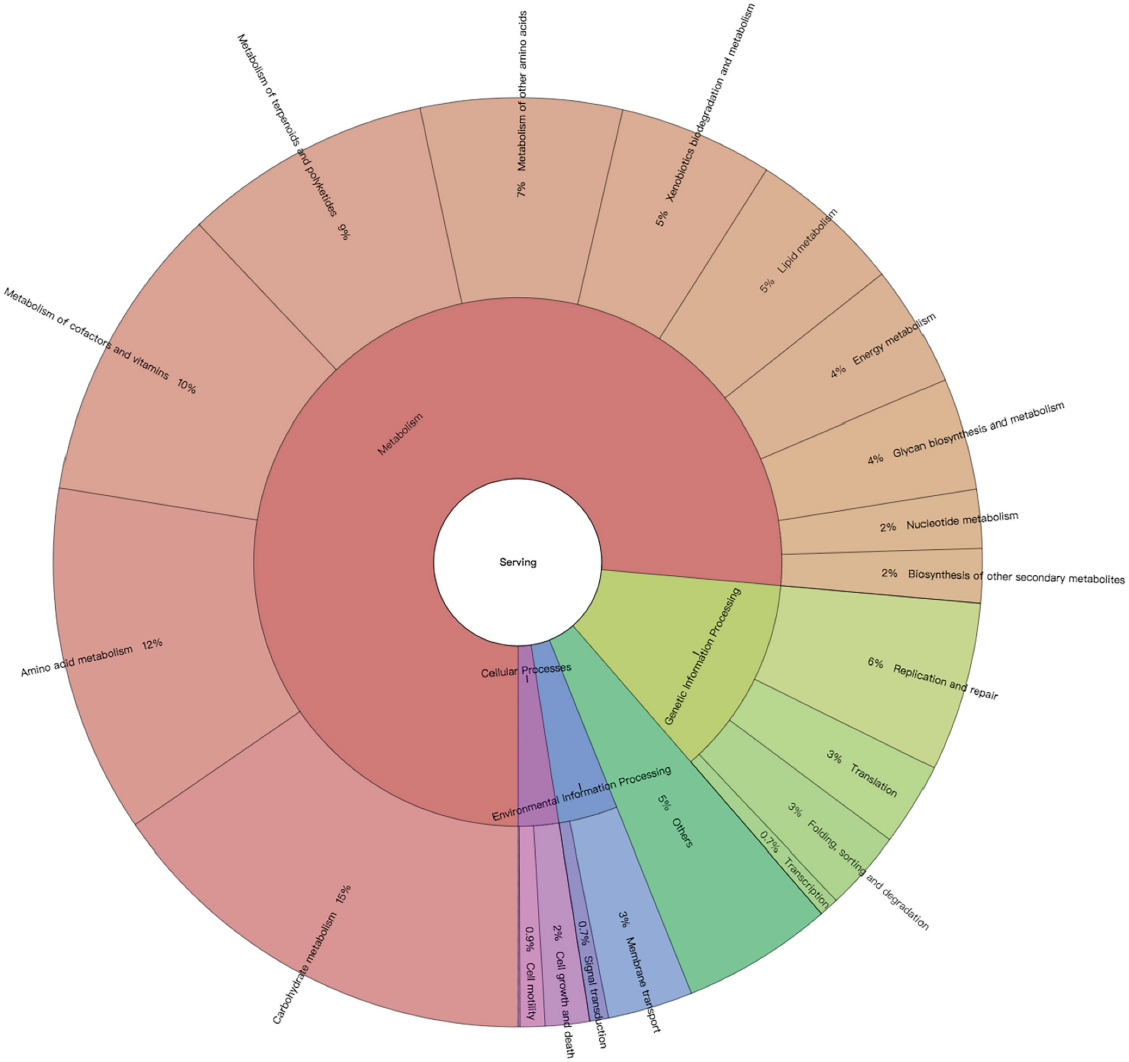
Profiles of neonatal calves’ colon microbiome. Microbial metabolic pathways based on their First and Second Level functions in the KEGG hierarchy.

Among all the generated metabolic pathways using HUMAnN2, 164 KEGG pathways were annotated from the colon metagenomes of neonatal calves. However, 25 pathways were removed as exogenous pathways. In total, 114 core pathways were identified in all the 24 colon samples with CPM > 5 (Supplementary Dataset 1), which belonged to four first-level KEGG functions, including “Cellular Processes” (2.47% ± 0.08%), “Environmental Information Processing” (3.63% ± 0.07%), “Genetic Information Processing” (12.23% ± 0.13%) and “Metabolism” (76.41% ± 0.18%). At the secondary level of KEGG hierarchy, 21 KEGG functions were identified, with “Carbohydrate metabolism” (15.43% ± 0.08%), “Amino acid metabolism” (12.13% ± 0.13%), “Metabolism of cofactors and vitamins” (10.39% ± 0.14%), “Metabolism of terpenoids and polyketides” (8.68% ± 0.13%), and “Metabolism of other amino acids” (7% ± 0.09%) being the top five functions (Figure 1). Meanwhile, the top five KEGG pathways were “ko01051: Biosynthesis of ansamycins” (3.94% ± 0.16%), “ko01055: Biosynthesis of vancomycin group antibiotics” (1.94% ± 0.09%), “ko00471: D-Glutamine and D-glutamate metabolism” (1.82% ± 0.03%), “ko00473: D-Alanine metabolism” (1.79% ± 0.04%), and “ko00290: Valine, leucine and isoleucine biosynthesis” (1.78% ± 0.04%; Supplementary Dataset 1).

### Taxonomic and functional composition of neonatal calves’ colon microbiome

**Figure 2:**
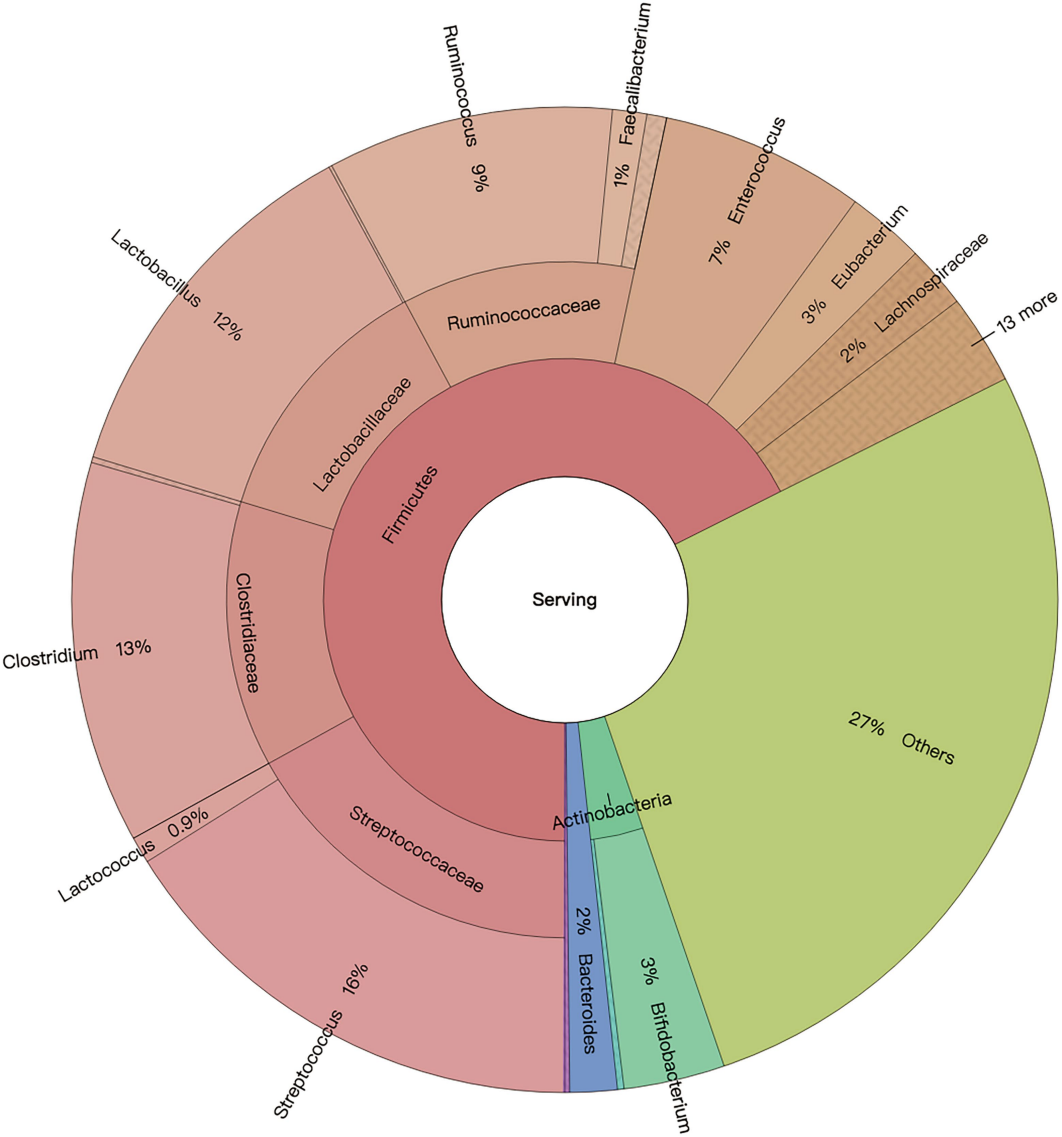
Colon bacterial composition of neonatal calves.

In general, four domains including bacteria, archaea, eukaryota, and viruses comprised the microbiome of 2-day-old calves. Regardless of the effect of colostrum feeding time treatment, bacteria was the predominant domain, with a relative abundance of 99.20% ± 0.09%, and archaea, eukaryotes, and viruses followed with relative abundances of 0.13 ± 0.01%, 0.09% ± 0.01%, 0.50% ± 0.09%, respectively (Supplementary Dataset 1). Bacterial and archaeal taxa with average relative abundance > 0.05%, and presented in more than 50% of the animals in at least one treatment, were defined as detected microbiota. For bacteria, colon microbiota consisted of nine bacterial phyla, including Firmicutes, Proteobacteria, Actinobacteria, Bacteroidetes, Fusobacteria, Spirochaetes, Cyanobacteria, Chloroflexi, and Thermotogae. Meanwhile, 35 bacterial families were detected, with *Enterobacteriaceae* (20.38% ± 3.17%), *Streptococcaceae* (16.96% ± 2.20%), *Lactobacillaceae* (14.79% ± 2.48%), *Clostridiaceae* (12.73% ± 1.15%), and *Ruminococcaceae* (11.47% ± 1.36%) being the top five most predominant bacterial families (Supplementary Dataset 1). Additionally, 441 bacterial genera were detected, with *Streptococcus* (16.08% ± 10.35%), *Clostridium* (12.54% ± 5.54%), *Lactobacillus* (12.37% ± 12.07%), *Ruminococcus* (9.38% ± 5.39%), and *Enterococcus* (6.71% ± 5.39%) being the top five bacterial genera (Figure 2).

Based on previous publications (Zhou et al., 2014; Nkamga et al., 2017; Song et al., 2021), the “non-gut archaeal taxa” were removed before microbial composition analysis. Taxonomic profiling revealed that sequences belonged to three archaeal phyla, 23 archaeal genera, and 20 archaeal species. The top five archaeal genera were *Methanobrevibacter* (15.89% ± 0.76%), *Methanosarcina* (11.83% ± 0.24%), *Methanocorpusculum* (10.59% ± 0.46%), *Methanococcus* (10.35% ± 0.65%), and *Methanosphaera* (4.51% ± 0.15%). Meanwhile, the top five archaeal species were *Methanocorpusculum labreanum* (11.34% ± 0.51%), *Methanobrevibacter smithii* (10.32% ± 0.63%), *Methanobrevibacter ruminantium* (6.63% ± 0.23%), *Methanosarcina acetivorans* (6.19% ± 0.16%), and *Methanosphaera stadtmanae* (4.81% ± 0.15%). It was found that 58% of sequences belonged to less abundant, unclassified, and non-gut Eukaryota phyla. In addition, viruses were unclassified at the phylum level.

### Effect of delayed colostrum feeding on colon microbiome composition

**Figure 3:**
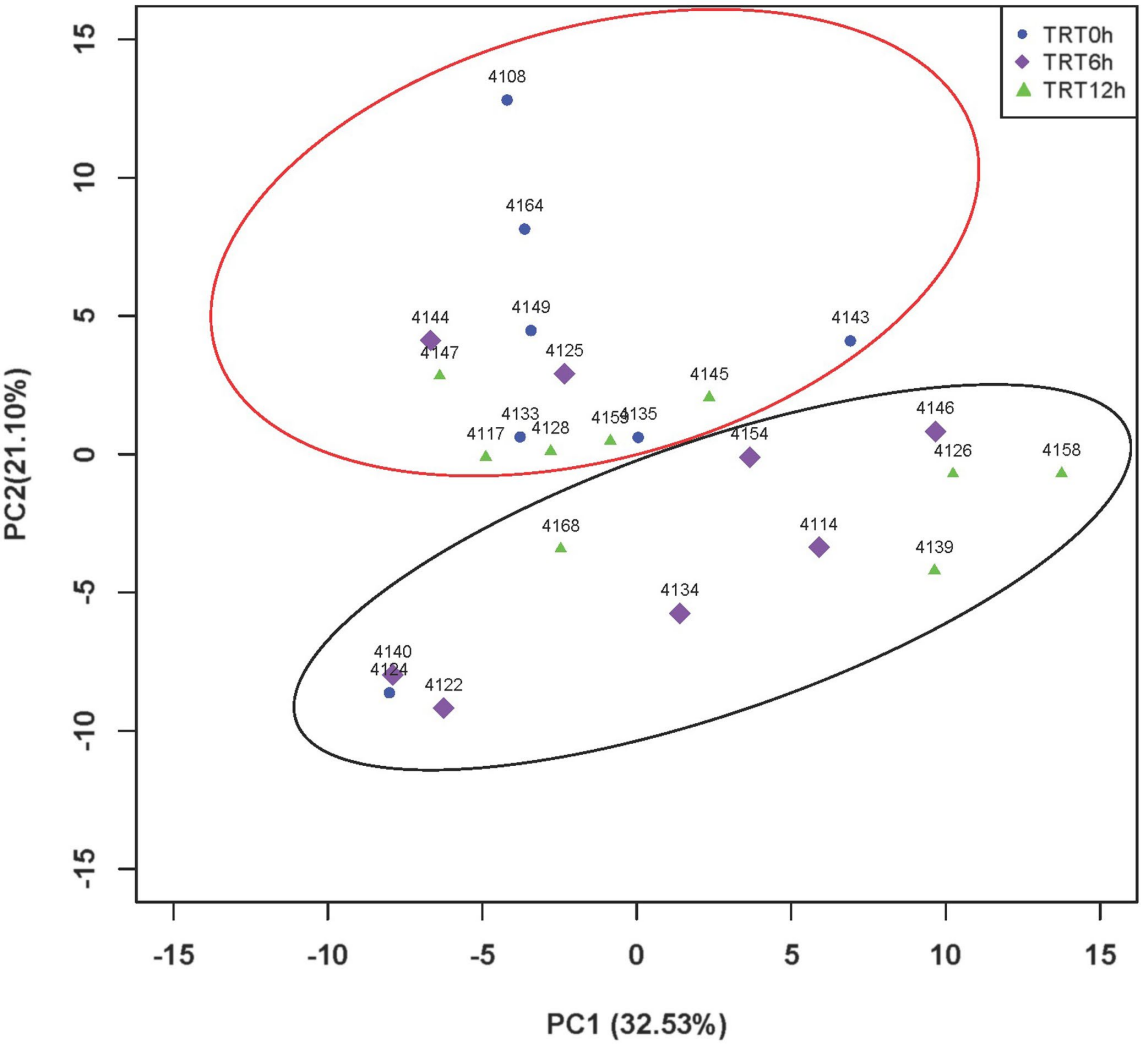
PCA plot of KEGG pathways among different treatments. Principal component analysis (PCA) plots of colon microbial metabolic Kyoto Encyclopedia of Genes and Genomes (KEGG) pathways among three colostrum feeding time treatments in neonatal dairy calves. TRT0h, TRT6h, and TRT12h indicate calves were fed colostrum at 45 min, 6 h, and 12 h after birth, respectively. PC1, principal component 1; PC2, principal component 2.

Colon bacterial community was compared using the Bray–Curtis dissimilarity matrices-based principal-coordinate analysis (PCoA), which revealed no clear separation among treatments (PERMANOVA, *p* = 0.65; Supplementary Figure 1A). Meanwhile, Shannon and Simpson indices were compared among treatments, and they were significantly higher in the calves from TRT6h and TRT12h groups compared to those in TRT0h group (Table 1). When the bacterial genera were compared at genus level, the relative abundance of four bacterial genera including *Parascardovia* (*P* < 0.01), *Scardovia* (*P* < 0.01), *Arthrobacter* (*p* = 0.01), and *Micrococcus* (*p* = 0.02) were found to be significantly higher in calves from TRT0h treatment, compared to calves in TRT6h and TRT12h treatment groups. In addition, the relative abundance of 14 bacterial groups was significantly higher in the colon digesta of the calves from TRT6h and TRT12h, compared to those calves in TRT0h group (Table 2). Additionally, Principal-coordinate analysis using Bray–Curtis dissimilarity matrices was also performed on the archaeal community, and no clear separated clusters were detected among different treatment groups (PERMANOVA, *p* = 0.80; Supplementary Figure 1B). Furthermore, the overall KEGG pathways in the colon digesta of calves were compared among three treatments using PCA analysis (Figure 3), and no clear separations among treatments (PERMANOVA, *p* = 0.11) were observed. Meanwhile, none of the KEGG pathways differed significantly among the three treatment groups.

**Table 1:** Effect of delayed colostrum feeding on colon bacterial diversity (mean ± SEM).

Diversity	Treatment			*P*-value
Matrix	TRT0h	TRT6h	TRT12h	
Shannon	2.39 ± 0.14^b^	2.80 ± 0.10^a^	2.76 ± 0.07^a^	0.02
Simpson	0.80 ± 0.02^b^	0.87 ± 0.01^a^	0.87 ± 0.01^a^	0.01

*P* < 0.05 means there is significant difference among groups; *P* < 0.1 means there is a tendency to be significant among groups.

^a,b^ Different superscripts within a row represent significance.

**Table 2:** Comparison of the relative abundance of bacterial genera among calves fed colostrum immediately after birth (TRT0h, *n* = 7), at 6 h after birth (TRT6h, *n* = 8) and at 12 h after birth (TRT12h, *n* = 9).

	Phylum	Family	Genus	Treatments			SEM	*P*-value^*^
				TRT0h	TRT6h	TRT12h		
Bacteria	Actinobacteria	*Bifidobacteriaceae*	*Parascardovia*	0.012^a^	0.003^b^	0.002^b^	0.002	0.00
			*Scardovia*	0.009^a^	0.003^b^	0.002^b^	0.001	0.00
		*Micrococcaceae*	*Arthrobacter*	0.026^a^	0.017^b^	0.013^b^	0.002	0.01
			*Micrococcus*	0.003^a^	0.002^b^	0.002^b^	0.001	0.02
			*Rothia*	0.031^a^	0.020^ab^	0.013^b^	0.004	0.02
	Firmicutes	*Leuconostocaceae*	*Weissella*	0.009^a^	0.006^ab^	0.004^b^	0.001	0.02
	Proteobacteria	*Aeromonadaceae*	*Tolumonas*	0.004^b^	0.007^a^	0.008^a^	0.001	0.01
		*Ferrimonadaceae*	*Ferrimonas*	0.001^b^	0.002^a^	0.002^a^	0.001	0.01
		*Vibrionaceae*	*Photobacterium*	0.011^b^	0.018^a^	0.018^a^	0.001	0.01
		*Vibrionaceae*	*Aliivibrio*	0.007^b^	0.010^a^	0.010^a^	0.001	0.02
		*Halomonadaceae*	*Chromohalobacter*	0.003^b^	0.005^a^	0.005^a^	0.001	0.02
		*Pseudomonadaceae*	*Pseudomonas*	0.034^b^	0.055^a^	0.055^a^	0.004	0.02
		*Vibrionaceae*	*Vibrio*	0.033^b^	0.051^a^	0.047^a^	0.003	0.02
		*Alcanivoracaceae*	*Kangiella*	0.001^b^	0.002^a^	0.002^a^	0.001	0.03
		*Enterobacteriaceae*	*Xenorhabdus*	0.004^b^	0.005^ab^	0.007^a^	0.001	0.03
		*Francisellaceae*	*Francisella*	0.006^b^	0.009^a^	0.008^a^	0.001	0.03
		*Rhodobacteraceae*	*Sulfitobacter*	0.001^b^	0.002^a^	0.001^b^	0.001	0.03
		*Shewanellaceae*	*Shewanella*	0.034^b^	0.054^a^	0.051^a^	0.004	0.03
		*Aeromonadaceae*	*Aeromonas*	0.011^b^	0.017^a^	0.018^a^	0.002	0.04
		*Desulfobulbaceae*	*Desulfotalea*	0.013^b^	0.027^a^	0.019^ab^	0.002	0.04
		*Oxalobacteraceae*	*Herminiimonas*	0.001^b^	0.002^a^	0.002^a^	0.001	0.04
	Tenericutes	*Acholeplasmataceae*	*Candidatus Phytoplasma*	0.002^b^	0.003^a^	0.003^a^	0.001	0.03
Archaea	Crenarchaeota	*Sulfolobaceae*	*Sulfolobus*	1.573^a^	0.884^b^	0.753^b^	0.151	0.04
	Euryarchaeota	*Methanocellaceae*	*Methanocella*	0.840^b^	0.959^ab^	1.149^a^	0.047	0.01

* *P*-values among the three colostrum treatment groups were obtained using non-parametric Kruskal–Wallis test statistical method. The significant difference between any two treatment groups was tested using Dunn’s test. The *P*-value was adjusted with Benjamini–Hochberg method for false discovery rate (FDR; Yoav and Yosef, 1995), with statistical significances declared at *P* < 0.05.

^a,b^ Different superscripts within a row represent significance.

### Effect of colostrum feeding time on SCFAs profiles

SCFAs concentrations were measured using GC, and acetate, propionate, and butyrate were detected. Generally, acetate had the highest concentration in the colon digesta. Butyrate concentration and its molar proportion were significantly higher in the colon digesta of calves in TRT0h compared to calves in TRT6h and TRT12h (*p* = 0.01). On the contrary, calves in TRT0h had lower acetate molar proportion compared to calves in TRT6h and TRT12h (Table 3).

**Table 3:** Short chain fatty acid (SCFA) concentrations (umol/g) and molar proportions among calves fed colostrum immediately after birth (TRT0h, *n* = 7), at 6 h after birth (TRT6h, *n* = 8) and at 12 h after birth (TRT12h, *n* = 9).

SCFAs	Treatments			SEM	*P*-value
	TRT0h	TRT6h	TRT12h		
Acetate^A^	11.75	13.31	9.26	1.25	0.46
Propionate^A^	1.98	2.51	2.19	0.37	0.88
Butyrate^A^	3.62^a^	0.90^b^	0.91^b^	0.40	0.01
Total SCFA^A^	17.36	16.71	12.36	1.65	0.41
Acetate^B^	0.68^b^	0.80^a^	0.76^a^	0.02	0.01
Propionate^B^	0.11	0.14	0.15	0.02	0.61
Butyrate^B^	0.21^a^	0.06^b^	0.10^b^	0.02	0.01

A Indicates differences in SCFA concentration.

B Indicates differences in the molar proportion of SCFA. Significance between treatment groups was declared at *p* < 0.05 and tendencies were declared at *p* < 0.10.

^a,b^ Different superscripts within a row represent significance.

### Individualized colon microbiome of 2-day-old calves

**Figure 4:**
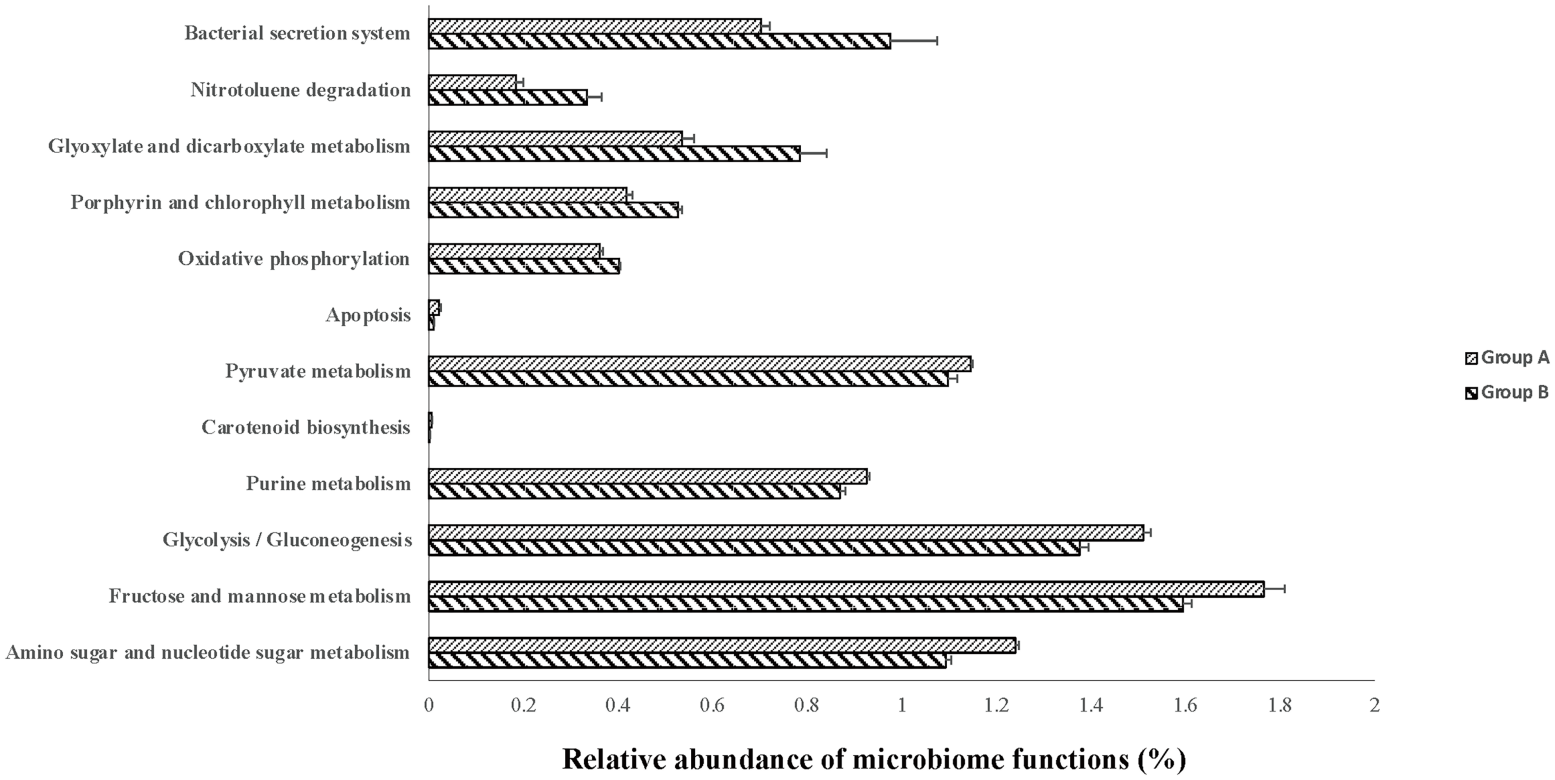
The relative abundance of varied second level Kyoto Encyclopedia of Genes and Genomes (KEGG) functions between calves with two type of colon metagenomes. Group A include five animals from the TRT12h treatment based on the principal component analysis plot (Figure 3) clearly separated from the other four animals in TRT12h treatment (group B).

Although the PCA did not show clear separation based on KEGG pathways among different colostrum feeding time treatments, it showed two major clusters regardless of treatments (PERMANOVA, *p* = 0.001; Figure 3). Cluster one consisted of 13 calves (TRT0h: *n* = 6, TRT6h: *n* = 2, TRT12h: *n* = 5), while cluster two included 11 calves (TRT0h: *n* = 1, TRT6h: *n* = 6, TRT12h: *n* = 4). Additionally, calves from the same treatment (TRT12h) were selected from clusters 1 and 2, defined as Group A (*n* = 5) and B (*n* = 4). Although these calves in Groups A and B were from the same colostrum feeding time treatment, we observed noticeable variations in the relative abundance of KEGG functions (Figure 4) through conducting the LEfSe analysis, and 12 differentially expressed KEGG pathways were identified (LDA score > 2 and *p* < 0.05) between Groups A and B. Specifically, seven KEGG pathways were more abundant in Group A, including “ko00520: Amino sugar and nucleotide sugar metabolism,” “ko00051: Fructose and mannose metabolism,” “ko00010: Glycolysis/Gluconeogenesis,” “ko00230: Purine metabolism,” “ko00906: Carotenoid biosynthesis,” “ko00620: Pyruvate metabolism,” and “ko04210: Apoptosis” (Figure 4). On the contrary, five KEGG pathways had higher relative abundance in Group B, including “ko00190: Oxidative phosphorylation,” “ko00860: Porphyrin and chlorophyll metabolism,” “ko00630: Glyoxylate and dicarboxylate metabolism,” “ko00633: Nitrotoluene degradation,” and “ko03070: Bacterial secretion system” (Figure 4).

Additionally, the taxonomic profiles were compared between Groups A and B. *Pediococcus* (*p* = 0.03), *Carnobacterium* (*p* = 0.02), *Oenococcus* (*p* = 0.02), *Staphylococcus* (*p* = 0.02), and *Thermoplasma* (*p* = 0.03) were more abundant in group A, while *Aeromonas* (*p* = 0.03), *Alcanivorax* (*p* = 0.03), *Edwardsiella* (*p* = 0.03), *Enterobacter* (*p* = 0.03), *Erwinia* (*p* = 0.03), *Escherichia* (*p* = 0.03), *Providencia* (*p* = 0.03), *Serratia* (*p* = 0.03), *Shigella* (*p* = 0.03), *Xenorhabdus* (*p* = 0.03), *Histophilus* (*p* = 0.03), *Photobacterium* (*p* = 0.03), *Candidatus Blochmannia* (*p* = 0.02), *Idiomarina* (*p* = 0.02), *Aggregatibacter* (*p* = 0.02), *Haemophilus* (*p* = 0.02), *Pseudomonas* (*p* = 0.02), *Shewanella* (*p* = 0.02), *Aliivibrio* (*p* = 0.02), and *Vibrio* (*p* = 0.02) had higher relative abundances in group B (Table 2).

### Colonic transcriptome profiles varied between calves with different microbiomes

In total, 62 DE genes were detected between groups A and B. Among them, 46 DE genes were upregulated in group A, while 16 genes were downregulated in group A (Supplementary Dataset 1). Further Venn Diagram analysis revealed that 13,992 genes were commonly expressed in the colon of groups A and B, and 189, 702 genes were uniquely expressed in groups A and B, respectively (Supplementary Figure 2). Meanwhile, the phenotypic parameters including the concentration of serum IgG, plasma GLP-1, GLP-2, insulin, acetate, propionate, and butyrate, as well as colon weight and length were compared between calves in groups A and B. However, only butyrate concentration was significantly different between groups A and B (A:1.47 ± 0.06 μmol/g; B:0.35 ± 0.04 μmol/g; *p* < 0.01).

### Correlation networks for identified differentially expressed genes, microbial taxa, and microbial functions between calves with different microbiomes

**Figure 5:**
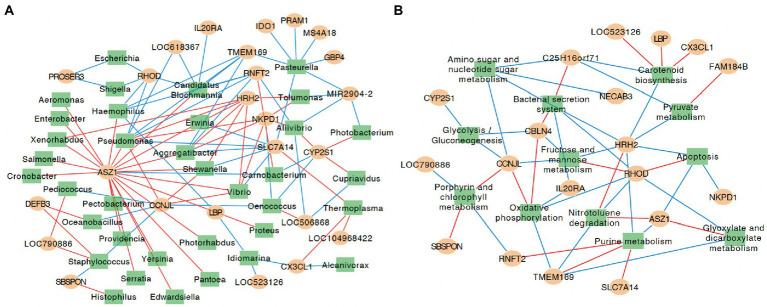
Correlation networks for identified differentially expressed genes, microbial taxa and microbial functions at KEGG pathway hierarchical level 3. **(A)** Correlation networks for identified differentially expressed genes and microbial taxa. **(B)** Correlation networks for identified differentially expressed genes and microbial functions at KEGG pathway hierarchical level 3. The yellow circles represent genes differentially expressed between groups A and B, and green rectangle represent the microbial taxa that were significantly different between groups A and B. The red lines indicate positive correlation, and blue lines indicate negative correlation; only correlations with rho values ≥ 0.8 and *p* ≤ 0.01 are shown. Data for genes/microbial taxa and functions that did not show a significant correlation are not shown.

The expression of DE genes between groups A and B was significantly correlated with the abundance of colon microbial taxa and functions. In total, the expression of 25 DE genes showed at least one correlation with 36 bacteria, and 19 DE genes were significantly correlated with 12 microbial functions (Figure 5). Generally, the expression of ankyrin repeat, SAM, and basic leucine zipper domain containing 1 (ASZ1), Cyclin J like (CCNJL), cytochrome P450, family 2, subfamily S, polypeptide 1 (CYP2S1), Histamine receptor H2 (HRH2), NTPase KAP family P-loop domain containing 1 (NKPD1), Ras homolog family member D (RHOD), Ring finger protein, transmembrane 2 (RNFT2), Solute carrier family 7 member 14 (SLC7A14), and Transmembrane protein 169 (TMEM169) was significantly correlated with no less than five bacterial taxa in the colon (Figure 5A). More specifically, the expression of *CCNJL* was negatively correlated with *Staphylococcus*, *Oenococcus*, *Pediococcus*, and *Oceanobacillus* (ρ < −0.8; *p* < 0.01), while it was positively correlated with *Pseudomonas*, *Shewanella*, and *Vibrio* (ρ values > 0.8; *p* < 0.01). The expression of *HRH2* was positively correlated with *Erwinia*, *Xenorhabdus*, *Pseudomonas*, *Aggregatibacter*, *Vibrio*, *Tolumonas*, and *Shewanella* (ρ values > 0.8; *p* < 0.01). The expression of *RHOD* was negatively correlated with *Candidatus Blochmannia, Pseudomonas, Escherichia, Shigella,* and *Haemophilus* (ρ values<− 0.8; *p* < 0.01). The expression of *TMEM169* was negatively correlated with *Candidatus Blochmannia, Pseudomonas, Pasteurella, Aggregatibacter,* and *Haemophilus* (ρ values< −0.8; *p* < 0.01).

Meanwhile, the expression of *CBLN4*, *CCNJL*, *HRH2*, and *RHOD* was closely correlated with no less than five colon microbial functions. The expression of *CCNJL* was negatively correlated with the relative abundances of amino sugar and nucleotide sugar metabolism, fructose and mannose metabolism, and Glycolysis/Gluconeogenesis (ρ values< −0.8; *p* < 0.01), and was positively correlated with oxidative phosphorylation and porphyrin and chlorophyll metabolism (ρ values > 0.8; *p* < 0.01). The expression of *HRH2* was negatively correlated with the relative abundances of amino sugar and nucleotide sugar metabolism, apoptosis, carotenoid biosynthesis, fructose and mannose metabolism, purine metabolism, and pyruvate metabolism (ρ values< −0.8; *p* < 0.01), and was positively correlated with the relative abundance of nitrotoluene degradation (ρ = 0.82, *p* < 0.01). The expression of *RHOD* was negatively correlated with the relative abundances of bacterial secretion system, glyoxylate and dicarboxylate metabolism, nitrotoluene degradation, and oxidative phosphorylation (ρ values< −0.8; *p* < 0.01), and was positively correlated with the relative abundance of apoptosis and fructose and mannose metabolism (ρ values > 0.8; *p* < 0.01; Figure 5B).

## Discussion

Similar to our previous finding of microbial composition in the ileum of 2-day-old calves (Song et al., 2021), bacteria, archaea, eukaryotes, and viruses constituted colon microbial microbiome, with bacteria being the most predominant. Compared with calves at 7-days old (Song et al., 2018), Firmicutes and Proteobacteria also dominated in the colon digesta at phylum level. However, a lower relative abundance of Bacteroidetes (20.81% ± 0.89% at D7, 1.71% ± 0.72% at D2) was detected at 2 days old. Meanwhile, the most abundant bacterial family (*Lactobacillaceae* with 22.36% ± 0.90% at D7; *Enterobacteriaceae* with 20.38% ± 3.17% at D2) and genera (*Lactobacillus* with 22.36% ± 1.37% at D7; *Streptococcus* with 16.08% ± 10.35% at D2) also differed between the two studies (Song et al., 2018). Such discrepancy may be caused by maternal factors, sequencing methods (amplicon sequencing vs. metagenomic sequencing), and colostrum feeding management strategies.

Although large intestinal microbiomes were mainly colonized by bacteria, the presence of archaea was revealed through metagenomic sequencing analysis. Similar to the predominant archaeal phyla in the human intestine at the first day of birth (Nkamga et al., 2017), Euryarchaeota (93.73%), Crenarchaeota (5.42%), and Thaumarchaeota (0.58%) were the most dominant archaea phyla in the colon of 2-day-old neonatal calves. This suggests that archaea may start to colonize the large intestine during the first 1–2 days after birth similar as the observation on their colonization in the rumen (Malmuthuge et al., 2019; Meale et al., 2021). Additionally, in agreement with our previous publication that *Methanobrevibacter*, *Methanosarcina*, and *Methanocorpusculum* were the three major archaeal genera in the small intestine using metagenomic sequencing analysis (Song et al., 2021), these three genera were also the predominant genera in the colon. Meanwhile, the archaeal species that related to methane synthesis accounted for 57.96% of all archaeal species, which may be closely associated with their functions in depleting H_2_ and carbohydrates degradation in the gut (Guindo et al., 2020). It was speculated that utilization of H_2_ for methane production could improve polysaccharide fermentation efficiency (Stams, 1994), which is beneficial for nutrient digestion in the large intestine, since the pre-weaning rumen has an undeveloped rumen. However, the functions of other archaeal species were not well studied and future studies are needed to explore the role that archaea may play in the colon of neonatal calves.

In addition, this study has provided the fundamental knowledge of colon microbial functions of 2-day-old dairy calves. The top five KEGG pathways at second level were all associated with nutrient metabolism. Moreover, “carbohydrate metabolism” (15.43% ± 0.08%) and “amino acid metabolism” (12.13% ± 0.13%) were the top two gene annotated KEGG pathways, which is similar to our previous findings of hindgut microbial functions of neonatal calves through functional prediction using 16S rRNA genes (Song et al., 2018). These results suggest that colon colonizers may play important roles in nutrient metabolism and energy harvest when the rumen is not well developed during the early neonatal period. Meanwhile, the top three metagenomic functions at level two of the KEGG pathways in the colon were similar to those in the ileum (Song et al., 2021), suggesting similar microbial functions regardless of gut sections of neonatal calves. Furthermore, two major third-level KEGG functions, including “biosynthesis of ansamycins” (3.94% ± 0.16%) and “biosynthesis of vancomycin group antibiotics” (1.94% ± 0.09%), were linked to antibiotics production, indicating that the early colon microbiome may inhibit the growth of other colonizers through producing antibiotics in scenarios of nutrient competition.

Notably, microbial diversity and richness were higher for calves that received colostrum at a 6 h and 12 h delay compared with calves fed colostrum immediately after birth, suggesting a complex microbiome establishment of the animals within these treatment groups. The observed changes of the colon digesta microbiome were driven by colostrum feeding time confounded with the total amount of nutrients that calves received over the 2-d study period. Overall, colostrum feeding time did not affect microbial functions, which may have been caused by high individual variation of animals among groups. Nevertheless, the relative abundances of some microbial taxa were influenced by different colostrum feeding time. The observed higher relative abundance of *Micrococcus* for TRT0h calves may be related to the higher concentration of butyrate in the colon digesta, since *Micrococcus* spp., such as *Micrococcus aerogenes,* could utilize colostrum glutamic acid for butyrate production (Horler et al., 1966). However, further studies are required to measure specific butyric acid-producing *Micrococcus* species using qPCR to confirm our speculation. Meanwhile, we speculate that increased relative abundance of *Aeromonas* genera in the 12-h delayed feeding group may lead to greater incidences of intestinal inflammation due to its classification as an opportunistic pathogen and its association with enteric lesions in cattle (Al-Mashat and Taylor, 1983). However, isolating specific pathogenic *Aeromonas* species is warranted to confirm such speculation. Of note, higher butyrate concentrations were observed in calves from the TRT0h group, which may promote intestinal development in these calves due to this microbial fermentation product’s ability to increase intestinal tissue mass and thickness (Tappenden et al., 2003), as well as enhance epithelial tight junctions, provide energy to epithelial cells (Lopetuso et al., 2013), and regulate immune system development (Arpaia et al., 2013). Therefore, we suggest that calves in TRT0h group may have the potential for a healthier gut. However, proof of this supposition needs to be further obtained by measuring intestinal permeability and histological examination.

Similar to previous studies reporting individualized small intestinal microbiome of neonatal calves (Malmuthuge et al., 2019; Song et al., 2021), the differences in colon microbiome were detected between individual animal groups (Groups A and B), even in animals within the same colostrum feeding treatment. Generally, the microbiota composition and functions of individual calf could be attributed to environmental, maternal, and host factors (Song et al., 2021). In detail, environmental factors include the farm environment (O'hara et al., 2020), the processing of colostrum and milk (fresh vs. pasteurized; Song et al., 2019), and feed additives in the milk (Badman et al., 2019; Wang et al., 2022). Maternal factors consist of the parity of the dam, maternal amniotic fluid (DiGiulio, 2012), placenta (Aagaard et al., 2014), as well as maternal colostrum (Yeoman et al., 2018). As such, several of these variables, including the bedding, disinfection strategy of the hutches, colostrum, milk replacer, and the dam parity in each colostrum feeding time treatment, were controlled in this study. Specifically, pasteurized colostrum was utilized for the calves in each treatment. Pasteurization would leave the nutritional and immunological factors intact but kill the vast diversity of microorganisms present in colostrum (Yeoman et al., 2018; Chen et al., 2021). Hence, all the calves were practically in the absence of maternally sourced and especially colostrum-sourced bacteria. Since environmental and management were controlled to be consistent for all calves, we suggest that host factors may be the main driver for the individualization of colon microbiota of neonatal calves. Host genetics have been reported to shape gut microbiomes of mice (Benson et al., 2010) and human infants (Goodrich et al., 2016). However, the extent to which the host may contribute to the individualized early colon microbiome colonization of neonatal calves is still unknown.

In this study, the difference of phenotypic traits, including DE genes, uniquely expressed genes in the colon tissue, and butyrate concentration in the colon digesta were detected between animals with two types of colon metagenomes, suggesting individual differences. Consistent with the phenotypic parameters, the variation in microbial taxa and functions were also detected between calves with two types of colon metagenomes. The microbial functions associated with nutrients metabolism, including amino sugar and nucleotide sugar metabolism, fructose and mannose metabolism, glycolysis/gluconeogenesis, purine metabolism, carotenoid biosynthesis, and pyruvate metabolism had higher levels of abundance in neonatal calves within group A than group B, highlighting that microbial nutrient metabolism functions are greatly affected by individual difference.

To further validate the extent by which individual host differences could affect the colon digesta microbiome, we explored how host gene expression may play a role. It is worth noting that significant correlations were identified between the expression levels of DE genes and the abundances of several microbial genus, such as Ras homolog family member D (*RHOD*) and the relative abundance of *Escherichia*. The enzyme rhodanese encoded by *RHOD* proved to be responsible for detoxifying hydrogen sulfide (H_2_S) in the colon (Picton et al., 2002), which was suggested to inhibit butyrate metabolism and cellular energy synthesis (Moore et al., 1997). Hence, the removal of H_2_S is beneficial for butyrate metabolism. Meanwhile, butyrate was reported to suppress *E. coli* colonization through increasing *Lactobacillus acidophilus* and *Bifidobacterium longum* adherence (Jung et al., 2022). Therefore, the expression of the *RHOD* may play a negative role in *E. coli* colonization, which is in agreement with our finding of a negative relationship between *RHOD* expression and Escherichia abundance. Similarly, multiple correlations were observed between the expression of host DE genes and the abundance of colon microbial pathways, which highlights the close interactions between the host and colon microbiome. Also, the identified correlations between the abundance of microbial nutrient utilization-related functions, such as pyruvate metabolism, fructose and mannose metabolism, amino sugar and nucleotide sugar metabolism, glycolysis/gluconeogenesis, and the expression of host DE genes implies host intestinal cell gene expression level may play important roles in regulating colon microbial metabolism. Meanwhile, other microbial functions, including bacterial secretion system and apoptosis were also affected by the different expression level of host genes. However, future studies through a combination of gene knockout technology and calf feeding trials are needed to elucidate the roles of the host on the intestinal microbiome.

## Conclusion

In conclusion, this study characterized the colon microbiome of 2-day-old dairy calves and how it altered when colostrum feeding time differed. The top five second-level KEGG pathways were all related to nutrients metabolism, with “carbohydrate metabolism” and “amino acid metabolism” being the top two gene annotated KEGG pathways, which suggested that colon colonizers may play important roles in nutrient metabolism and energy utilization during the early neonatal period. In general, delaying colostrum feeding did not influence the overall colon microbiome of 2-day-old dairy calves at both the taxonomic and functional levels, and the short duration of the animal trial could be one of the reasons. Long-term effects of delaying first colostrum feeding to colon microbiome are needed in the further study. Individualized colon microbiome profiles were observed regardless of colostrum feeding time treatment. This suggests that host factors may play an important role in shaping early life colon microbiome of the dairy calves. The correlation between identified DE genes, microbial taxa and microbial functions between calves with varied colon microbiomes further proved the effect of host on colon microbiome. However, further studies are encouraged to explore the extent by which the host may have an effect on the initial colonization of the colon microbiome by gene knockout technology of the DE genes.

## Data availability statement

The data presented in the study are deposited in the MG-RAST repository, accession number: https://www.mg-rast.org/linkin.cgi?project=mgp82806.

## Ethics statement

The animal study was reviewed and approved by The Livestock Care Committee of the University of Alberta (AUP00001595).

## Author contributions

YS participated in animal sampling and metagenomic library construction. YS and SW contributed to data analysis and writing and revision of the manuscript. FL helped with data processing. AF-T helped with colon digesta sample collection. ZH provided the original data of colon metatranscriptomic data. MS participated in the experiment design and paper revise. LG participated in the study design and revised the manuscript. All authors contributed to the article and approved the submitted version.

## Funding

This work was supported by Program for Young Talents of Science and Technology in Universities of Inner Mongolia Autonomous Region (NJYT22053; Inner Mongolia, China), the Natural Science Foundation of Inner Mongolian (2022LHQN03009, 2020BS03028; Inner Mongolia, China), Doctoral Funding of the Inner Mongolia University for the Nationalities (BS583, BS584; Inner Mongolia, China), and Alberta Livestock and Meat Agency Ltd. (Edmonton, Canada), Alberta Milk (Edmonton, Canada), the Saskatoon Colostrum Co. Ltd. (Saskatoon, Canada), the Natural Sciences and Engineering Research Council (NSERC) of Canada (Discovery grant; Ottawa, Canada). The authors declare that this study received funding from the Saskatoon Colostrum Co. Ltd. (Saskatoon, Canada). The funder was not involved in the study design, collection, analysis, interpretation of data, the writing of this article, or the decision to submit it for publication.

## Conflict of interest

The authors declare that the research was conducted in the absence of any commercial or financial relationships that could be construed as a potential conflict of interest.

## Publisher’s note

All claims expressed in this article are solely those of the authors and do not necessarily represent those of their affiliated organizations, or those of the publisher, the editors and the reviewers. Any product that may be evaluated in this article, or claim that may be made by its manufacturer, is not guaranteed or endorsed by the publisher.
