# Supplementary material for: Metagenomic analysis provides bases on individualized shift of colon microbiome affected by delaying colostrum feeding in neonatal calves
Source: Front Microbiol. 2022 Nov 1;13:1035331. doi: 10.3389/fmicb.2022.1035331 (PMC9664197; doi:10.3389/fmicb.2022.1035331)
Supplement: Supplementary file 2 [file Presentation_1.PPTX]

## Slide 1
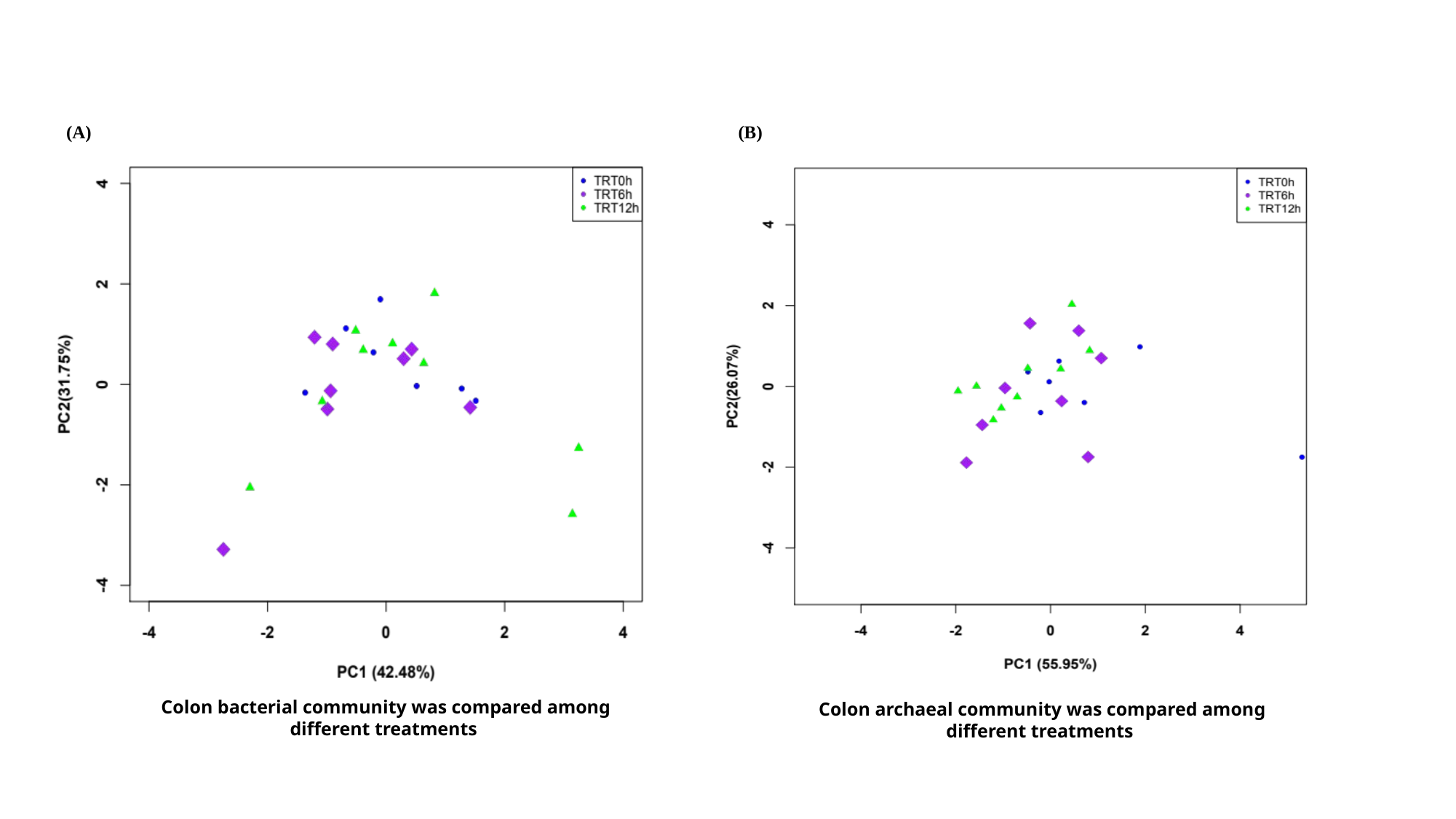

(A)
(B)
Colon bacterial community was compared among different treatments
Colon archaeal community was compared among different treatments
