# Supplementary material for: Metagenomic analysis provides bases on individualized shift of colon microbiome affected by delaying colostrum feeding in neonatal calves
Source: Front Microbiol. 2022 Nov 1;13:1035331. doi: 10.3389/fmicb.2022.1035331 (PMC9664197; doi:10.3389/fmicb.2022.1035331)
Supplement: Supplementary file 3 [file Presentation_2.PPTX]

## Slide 1
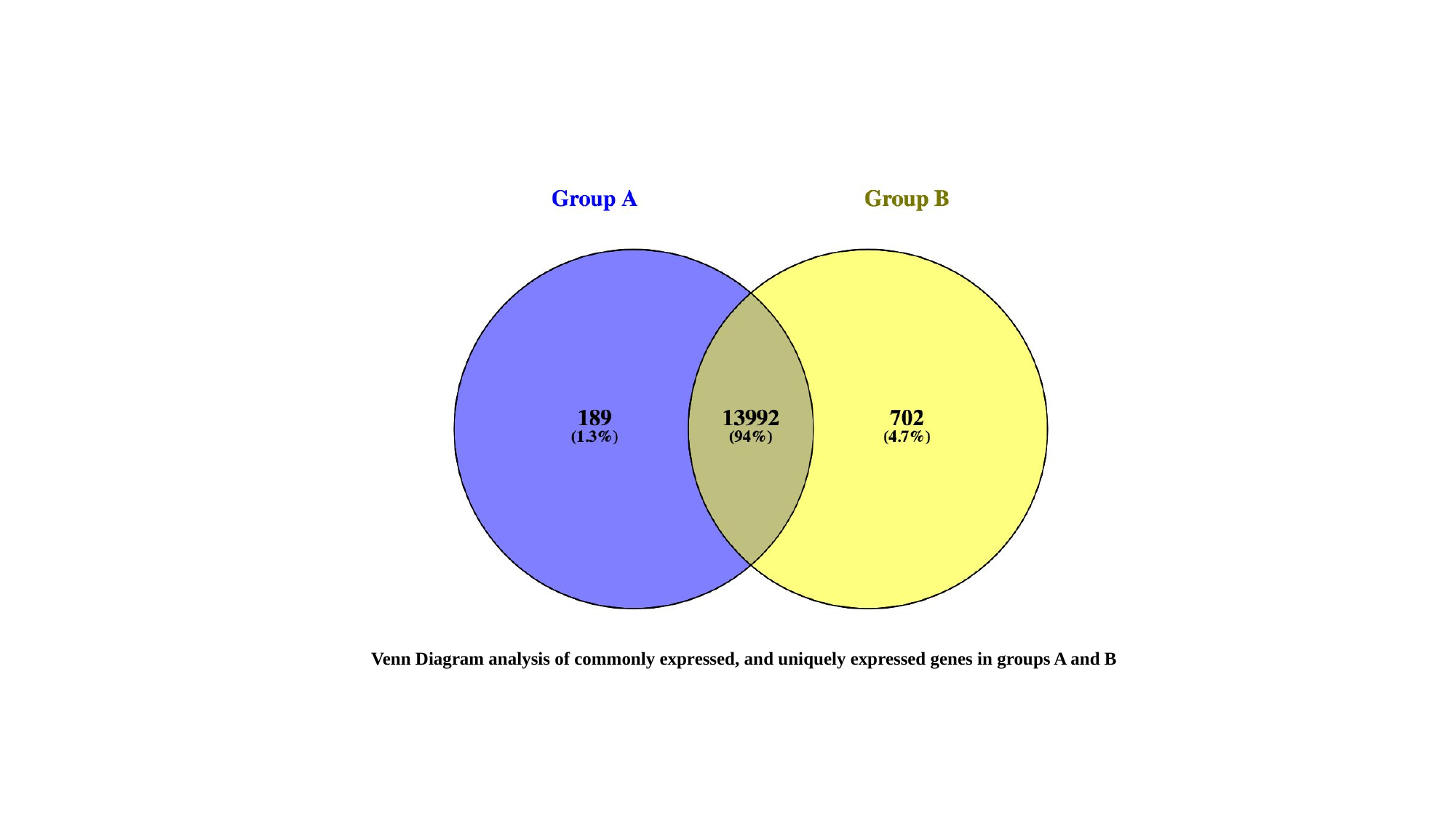

Venn Diagram analysis of commonly expressed, and uniquely expressed genes in groups A and B
